# The emerging role of glycine receptor α2 subunit defects in neurodevelopmental disorders

**DOI:** 10.3389/fnmol.2025.1550863

**Published:** 2025-02-11

**Authors:** Sean D. Fraser, Robert J. Harvey

**Affiliations:** ^1^School of Health, University of the Sunshine Coast, Maroochydore, QLD, Australia; ^2^National PTSD Research Centre, Thompson Institute, University of the Sunshine Coast, Birtinya, QLD, Australia

**Keywords:** autism spectrum disorder, cortical neuronal migration, epilepsy, glycine receptor, *Glra2* knockout mice, GlyR α2 subunit, intellectual disability, neurodevelopmental disorders

## Abstract

Rare neurodevelopmental disorders (NDDs) are one of the most significant unmet challenges in healthcare due to their lifelong nature, high management costs, and recurrence within families. This review will focus on newly-emerging genetic forms of NDDs resulting from variants in the glycine receptor (GlyR) α2 subunit gene. Studies using *Glra2* knockout mice have convincingly demonstrated that GlyR α2 is essential for cortical interneuron migration and progenitor homeostasis. Genetic inactivation of GlyR α2 impairs the capacity of apical progenitors to generate basal progenitors, resulting in an overall reduction of projection neurons in the cerebral cortex. As a result, microcephaly is observed in newborn *Glra2* knockout mice, as well as defects in neuronal morphology, increased susceptibility to seizures, and defects in novel object recognition, motor memory consolidation, righting reflexes, novelty-induced locomotion in the open field test, and motivational reward tasks. Consistent with these findings, we and others have identified missense variants and microdeletions in the human GlyR α2 subunit gene (*GLRA2*) in individuals with autism spectrum disorder (ASD), developmental delay (DD) and/or intellectual disability (ID), often accompanied by microcephaly, language delay and epilepsy. In this review, we highlight the critical role of the GlyR α2 subunit revealed by knockout mice and our current understanding of GlyR α2 pathomechanisms in human NDDs. Finally, we will consider the current gaps in our knowledge, which include: (i) Limited functional validation for GlyR α2 missense variants associated with human NDDs; (ii) The lack of *gain-of-function* GlyR α2 mouse models; (iii) Our limited knowledge of GlyR α2 interacting proteins. We also highlight potential future developments in the field, including routes to personalized medicines for individuals with GlyR α2 mutations.

## Introduction

Glycine receptors (GlyRs) are recognized as mediators of fast inhibitory synaptic neurotransmission in the spinal cord and brainstem (Breitinger and Becker, 2002), in addition to the retina and inner ear (Wässle et al., 2009; Buerbank et al., 2011). GlyRs are members of the pentameric cys-loop ligand-gated ion channel family, with other members including the nicotinic acetylcholine receptor (nAChR), serotonin type 3 receptor (5-HT_3_R), and *γ*-aminobutyric acid type A receptor (GABA_A_R) (Lynch, 2004, 2009). Members of this superfamily share a common pentameric, symmetrical configuration of subunits arranged around a central, ion-conducting pore that traverses the cell membrane (Du et al., 2015; Huang et al., 2015; Yu et al., 2021; Zhu and Gouaux, 2021; Gibbs et al., 2023). Each GlyR subunit comprises an N-terminal signal peptide (SP); a large, agonist-binding extracellular domain (ECD); four membrane-spanning domains (TM1-TM4) connected by loops of varying length and a short extracellular C-terminus. Five evolutionarily conserved, yet distinct GlyR subunits have been described in humans and rodents: the GlyR α1-α4 and β subunits. These subunits assemble into pentameric homomeric α or heteromeric αβ receptors. The stoichiometric configuration of heteromeric GlyRs has been a matter of contention. However, the recent analysis of GlyRs via cryo-electron microscopy suggests that heteromeric GlyRs have an invariant 4α:1β configuration (Yu et al., 2021; Zhu and Gouaux, 2021). Given their evolutionarily conserved nature, the GlyR α subunits share a high degree of sequence identity (≥80%) (Grenningloh et al., 1990; Matzenbach et al., 1994). The region featuring the least sequence similarity is the TM3-TM4 intracellular domain (ICD), which mediates interactions with accessory proteins, such as gephyrin (Prior et al., 1992; Meyer et al., 1995), collybistin (Breitinger et al., 2021) and syndapin I (Del Pino et al., 2014; Troger et al., 2022), as well as providing a site for post-translational modification, including phosphorylation and ubiquitination (Harvey K. et al., 2004; Langlhofer and Villmann, 2016).

GlyR α and β subunits display distinct spatiotemporal expression profiles within the mammalian CNS. In particular, GlyR α2 is widely expressed in brain and spinal cord during embryonic development and early postnatal life, but is replaced by GlyR α1 and α3 during postnatal maturation (Becker et al., 1988; Akagi et al., 1991; Malosio et al., 1991). The GlyR α1 subunit is known for its role in providing fast synaptic inhibition within motor reflex pathways of the spinal cord (Lynch, 2009). These GlyRs are hypothesized to comprise a heteromeric α1β configuration, given that gephyrin is necessary for the postsynaptic localization of GlyRs and only the β subunit is capable of interacting with this scaffolding protein via the gephyrin E domain (Meyer et al., 1995; Rees et al., 2003; Harvey K. et al., 2004; Sola et al., 2004). The importance of heteromeric α1β GlyRs in tempering the excitability of motoneurons is evidenced by studies of the rare neurological disorder startle disease/hyperekplexia (Harvey et al., 2008; Schaefer et al., 2022), which is caused by mutations within the corresponding genes: *GLRA1* and *GLRB* (Shiang et al., 1993; Rees et al., 2002), *SLC6A5* encoding the presynaptic glycine transporter GlyT2 (Rees et al., 2006; Carta et al., 2012; Giménez et al., 2012) and *SLC7A10* encoding Asc-1, an alanine-serine-cysteine transporter (Drehmann et al., 2023). Numerous spontaneous mouse mutants with defects in the *Glra1* and *Glrb* subunit genes are also known, including *spastic*, *spasmodic*, *oscillator*, *cincinatti*, *nmf11* and *shaky*, which have proven to be outstanding models for the study of anxiety and startle phenotypes (Schaefer et al., 2022). GlyR α3 is found in the spinal cord dorsal horn where pain-sensing nerve fibers arrive from the periphery (Harvey R. J. et al., 2004), as well as in neurons within the brainstem pre-Bötzinger complex that control breathing (Manzke et al., 2010). In both regions, GlyR α3 is modulated by different G-protein-coupled receptors that lead to downstream changes in the phosphorylation state of GlyR α3. The generation and analysis of knockout and knock-in mice has revealed roles for the GlyR α3 subunit in inflammatory pain sensitization (Harvey R. J. et al., 2004; Werynska et al., 2021), rhythmic breathing (Manzke et al., 2010), auditory nerve function (Dlugaiczyk et al., 2016), as well as ethanol-related addictive behaviors (Blednov et al., 2015; San Martin et al., 2021). Lastly, GlyR α4 impacts embryonic development, litter sizes, startle responses, and anxiety-like behaviors in mice (Nishizono et al., 2020; Darwish et al., 2023) but is a pseudogene in humans (Leacock et al., 2018).

**Figure 1:**
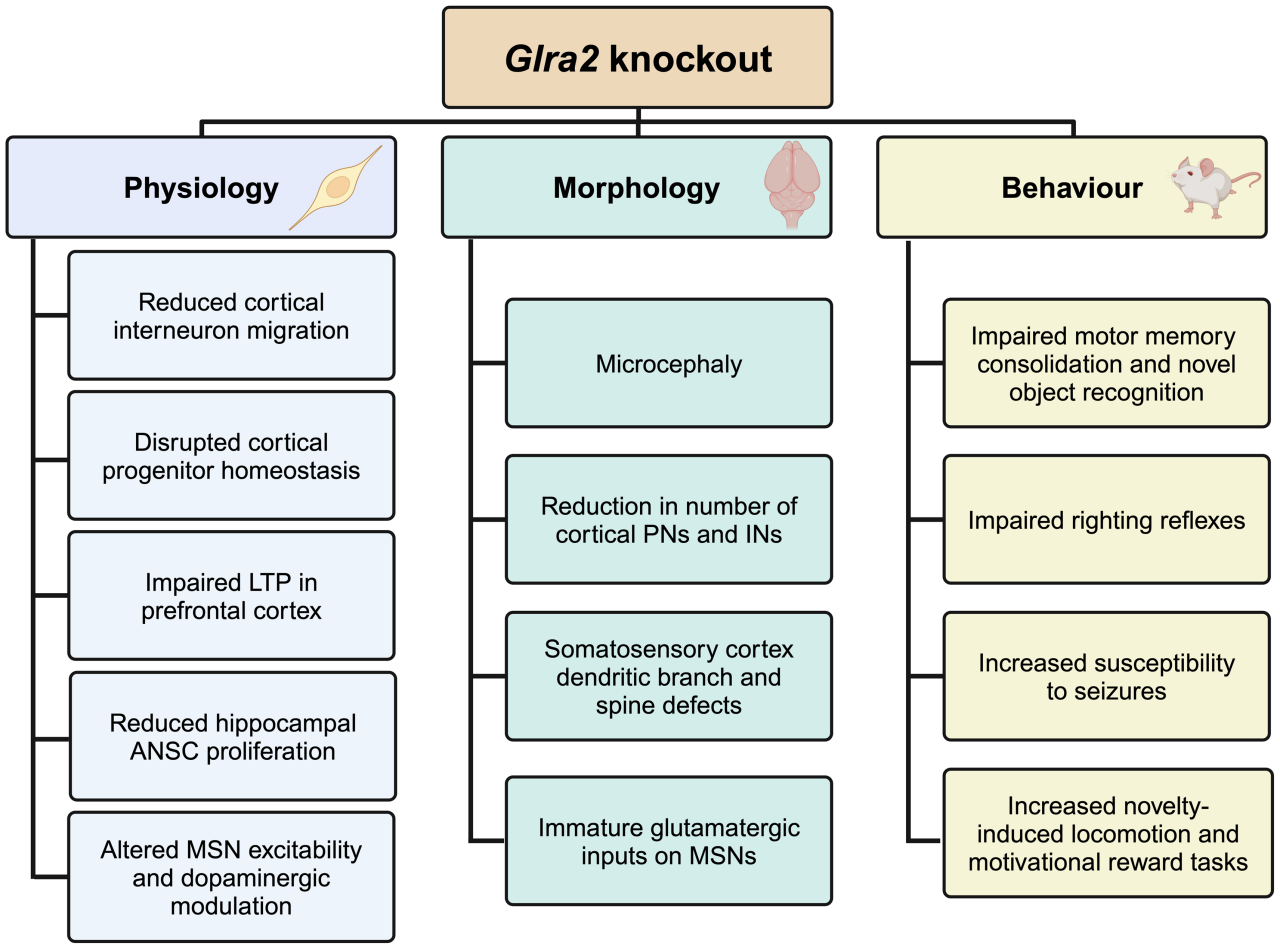
Summary of the physiological, morphological and behavioral phenotypes observed in *Glra2* knockout mice. KO, knockout; LTP, long-term potentiation; ANSC, adult neural stem cell; MSN, medium spiny neuron; PN, projection neuron; IN, interneuron. Created in https://BioRender.com.

This review will focus on the biological role of the GlyR α2 subunit, highlighting a series of studies using newly-developed GlyR α2 subunit knockout mice that linked this receptor subtype to multiple defects in neuronal physiology, morphology and behaviors (Figure 1). This led to the subsequent discovery of a microdeletion and missense variants in the human GlyR α2 subunit in cases of autism spectrum disorder, intellectual disability and neurodevelopmental disorders. We highlight fundamental concepts, knowledge gaps and potential future developments in the field, including a roadmap to personalized medicines for individuals with pathogenic GlyR α2 mutations.

## Biological roles of the GlyR α2 subunit

The GlyR α2 subunit was initially designated as the ‘neonatal isoform’ (49 kDa) of the GlyR, distinguishable from the ‘adult isoform’ (α1, 48 kDa) by virtue of molecular weight and an embryonic/neonatal expression pattern in rodents (Becker et al., 1988; Akagi et al., 1991). GlyR α2 shows ubiquitous expression throughout the developing CNS (Kuhse et al., 1991; Malosio et al., 1991) reaching peak expression within the first few weeks after birth and declining in postnatal stages. GlyRs containing the α2 or α3 subunits exhibit significantly larger main state single-channel conductances compared to those comprised of the α1 subunit (Bormann et al., 1993) due to a single non-conserved alanine to glycine substitution at the intracellular end of the pore-lining TM2 domain (Bormann et al., 1993). The rapid short-burst activity mediated by GlyR α1β channels in response to low glycine concentrations makes them well-suited to mediating inhibitory synaptic currents (Krashia et al., 2011; Zhang Y. et al., 2015). Conversely, the currents mediated by GlyR α2 and α2β channels are characterized by significantly slower rise and decay times (Mangin et al., 2003; Zhang Y. et al., 2015). Mangin et al. (2003) concluded that the slow kinetic properties exhibited by GlyR α2 channels are ill-suited to the phasic neurotransmission, but rather better equipped for tonic activation at non-synaptic sites. In line with these extrasynaptic activation kinetics, the GlyR α2 subunit was initially assigned a key role in synaptogenesis (Kirsch and Betz, 1998; Levi et al., 1998). Levi et al. (1998) demonstrated that the chronic treatment of cultured spinal cord neurons from E14 rats with the GlyR antagonist strychnine impeded the formation of postsynaptic GlyR clusters. The inhibition of GlyR activation by strychnine affected the ability of GlyR clusters to form within the somatodendritic membrane (Levi et al., 1998). Kirsch and Betz (1998) went a step further and deduced that GlyR activation triggers synaptogenesis via membrane depolarization (i.e., Cl^−^ efflux), which facilitates the opening of L-type Ca^2+^ channels. The resulting Ca^2+^ influx causes the accumulation of gephyrin at the membrane, ‘trapping’ the newly formed GlyR clusters opposite presynaptic terminals (Kirsch and Betz, 1998). Flint et al. (1998) were the first to propose a potential role for GlyR α2 in neocortical development, as the main conductance states of GlyRs expressed by embryonic and neonatal cortical neurons resembled α2 channels. GlyR activation in immature cortical neurons stimulated the synaptic release of GABA from GABAergic interneurons via a rise in intracellular Ca^2+^ concentrations (Flint et al., 1998). They also hypothesized that the GlyR-mediated increase in membrane depolarization – resulting in increased intracellular Ca^2+^ − could play a role in neurogenic processes, such as cellular migration and differentiation (Flint et al., 1998). However, proof of this concept required the generation of GlyR α2 knockout mice.

## Lessons learnt from GlyR α2 mouse models

### GlyR α2 defects in retinal signaling pathways and vision

GlyR α2 knockout mouse models have provided unrivaled insights into the biological role of the GlyR α2 subunit. The first GlyR α2 knockout mouse model was generated by Young-Pearse et al. (2006), who replaced exons 6 and 7 of the mouse *Glra2* locus with a phosphoglycerate kinase I promoter neomycin phosphotransferase gene cassette, resulting in a frameshift and loss of part of the ECD and TM1-TM4. This model was generated to study rod photoreceptors in the neonatal retina, which were reduced after transient knockdown of GlyR α2 using siRNA (Young and Cepko, 2004). The hypothesis was that GlyR α2 activation induced retinal progenitor cells to exit mitosis and generate rods within the photoreceptor layer (Young and Cepko, 2004). Curiously, this retinal phenotype was not observed in the GlyR α2^ΔEx6–7^ knockout mouse model as no morphological, molecular or electroretinogram differences were reported in neonatal retinae (Young-Pearse et al., 2006). However, later studies using knockout mice have implicated GlyR α2 in crossover inhibition between ON and OFF retinal pathways (Nobles et al., 2012), as well as in the modulation of the receptive field surround of OFF retinal ganglion cells (Zhang C. et al., 2015). More recently Tian et al. (2023) examined ocular phenotypes using a novel *Glra2* mouse knockout line generated by removing exon 2 using CRISPR/Cas9 technology, resulting in a frameshift and loss of the majority of the ECD and TM1-TM4. Surprisingly, Tian et al. (2023) recorded a significant reduction in the formation of rod photoreceptors in GlyR α2^ΔEx2^ knockout retinae compared to wild-type littermates, in addition to deficits in rod pathway transduction as evidenced by a reduced dark-adapted electroretinography response. Other ocular defects observed in the GlyR α2^ΔEx2^ knockouts were reduced visual acuity and corneal thickness, and mice were more myopic than their wild-type littermates (Tian et al., 2023). The reasons for the phenotypic differences in retinal phenotypes between GlyR α2^ΔEx2^ and GlyR α2^ΔEx6–7^ knockout lines remain unclear.

### GlyR α2 defects in corticogenesis

**Figure 2:**
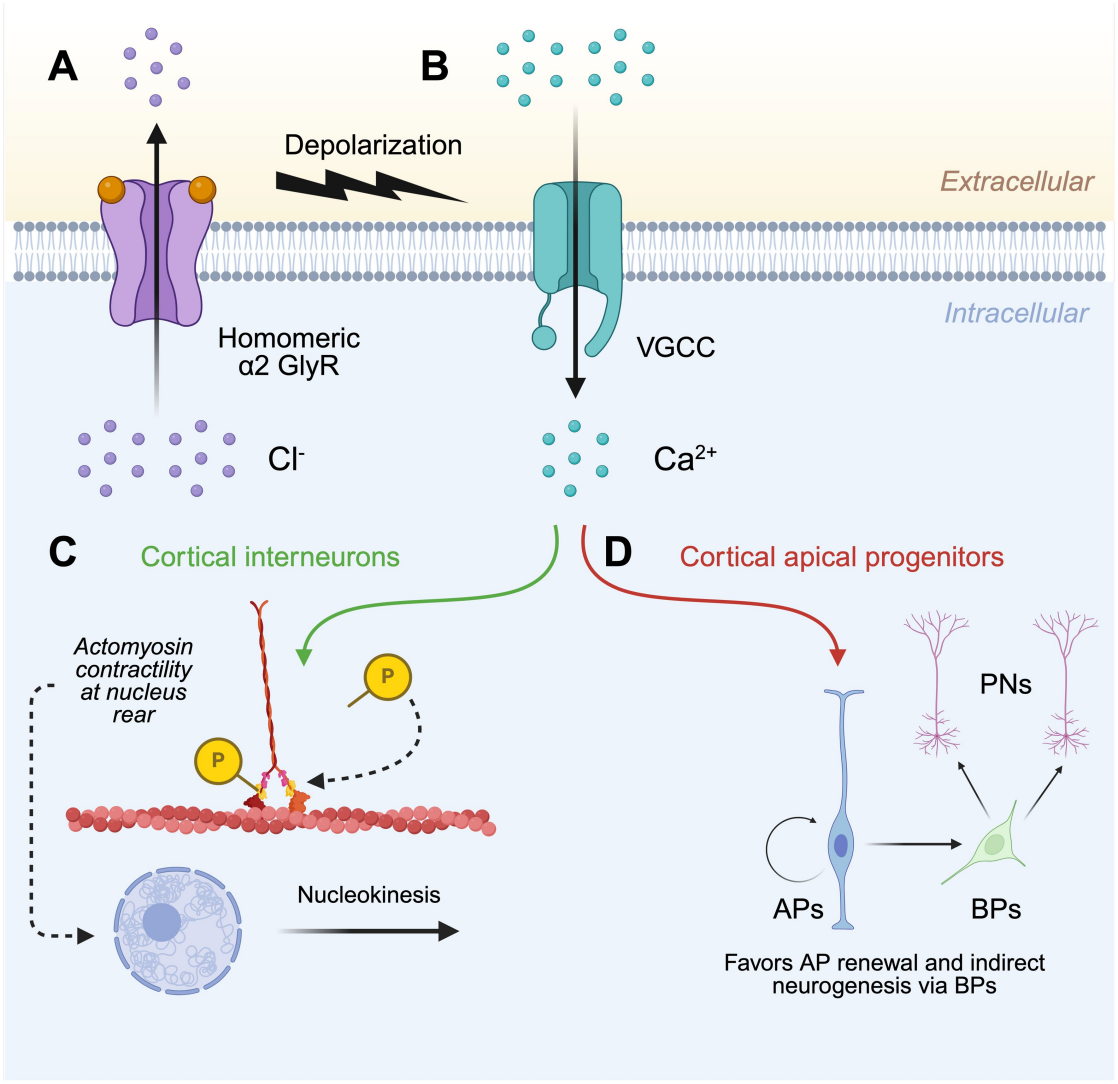
Model for the role of GlyR α2 in cortical development. **(A)** The activation of homomeric α2 GlyRs in the developing cerebral cortex causes membrane depolarization by facilitating Cl^−^ efflux. **(B)** Membrane depolarization triggers the opening of voltage-gated Ca^2+^ channels and subsequent Ca^2+^ influx, which within **(C)** cortical interneurons promotes nucleokinesis through the phosphorylation of myosin light chain, resulting in the accumulation and contraction of actomyosin at the rear of the nucleus. **(D)** Ca^2+^ influx also ensures the proper generation of projection neurons via the homeostatic self-renewal and differentiation of cortical apical progenitors. GlyR, glycine receptor; VGCC, voltage-gated Ca^2+^ channel; AP, apical progenitor; BP, basal progenitor; PN, projection neuron. Created in https://BioRender.com.

In 2013, Harvey and Dear generated a novel GlyR α2 knockout line (GlyR α2^ΔEx7^), employing the *Cre*-Lox gene targeting system to excise exon 7 of *Glra2*, resulting in the loss of TM1-TM3, and TM4 via a frameshift (Avila et al., 2013). Initially, the GlyR α2^ΔEx7^ knockout mouse line was used to investigate the role of GlyR α2 in corticogenesis (Avila et al., 2013; Avila et al., 2014). Firstly, a range of observational and functional assays including immunolabeling, Western blot analyses and electrophysiological recordings established that functional homomeric GlyR α2 channels were found in interneurons of the embryonic cortex (Avila et al., 2013). Acute inhibition of these channels via strychnine application to cultured embryonic brain slices decreased the migration velocity and nucleokinesis frequency of cortical interneurons (Avila et al., 2013). These observations were also reported in the embryonic brains of GlyR α2^ΔEx7^ knockout mice, as interneurons traveling in the subventricular zone (SVZ) stream exhibited a reduction in their migration velocity and frequency of nuclear translocation (Avila et al., 2013). Building upon the observations of Flint et al. (1998) that GlyR activation in the developing cerebral cortex triggered depolarization-mediated Ca^2+^ influx, Avila et al. (2013) reported a decreased velocity of interneuron migration in the presence of N-type and L-type voltage-gated Ca^2+^ channel blockers (omega-conotoxin and calciseptin). This suggested an integral role for Ca^2+^ influx – triggered by GlyR activation – in this process. Downstream of Ca^2+^ influx, it was noted that treatment with a myosin light chain (MLC) kinase blocker (ML-7) reduced migration velocity and nuclear translocation as was observed in the GlyR α2^ΔEx7^ knockout or upon strychnine application to cultured embryonic brain tissue (Avila et al., 2013). Together, these results suggested that activation of homomeric GlyR α2 triggers depolarization-mediated Ca^2+^ influx causing the phosphorylation of MLC and activation of the myosin II complex. This results in the accumulation and contraction of actomyosin fibers at the rear of the cortical interneuron nucleus, thus promoting nucleokinesis (Figure 2; Avila et al., 2013).

Avila et al. (2014) next sought to examine a potential role of GlyR α2 in the generation of cortical projection neurons. Immunolabeling and electrophysiological recordings demonstrated prolific expression of GlyR α2 throughout the embryonic cortex (Avila et al., 2014). In particular, GlyR α2 localized to apical progenitor (AP) and basal progenitor (BP) cells of the ventricular and subventricular zones, respectively (Avila et al., 2014). Gross morphological analysis of newborn GlyR α2^ΔEx7^ knockout mice revealed microcephaly, with a significant thinning of the cerebral cortical wall (Avila et al., 2014). Given that a reduction in cortical interneurons alone could not account for the significant reduction in cortical wall thickness, one hypothesis was that this resulted from poor survival of newborn neurons and their progenitors (Avila et al., 2014). Comparison of GlyR α2^ΔEx7^ knockout mice with controls revealed a depletion of BPs, and later a progressive depletion of APs (Avila et al., 2014). APs from GlyR α2^ΔEx7^ knockout mice showed a propensity for direct neurogenesis, largely bypassing the generation of BPs – a cell population that acts as progenitors for the formation of projection neurons (Avila et al., 2014). Furthermore, cortical progenitor cell cycle exit was increased in GlyR α2^ΔEx7^ knockout embryos, suggesting that loss of GlyR α2 causes the premature differentiation of BPs (Avila et al., 2014). Due to this predisposition for APs to undergo direct neurogenesis and BPs to prematurely differentiate, the progressive exhaustion of the cortical progenitor pool in GlyR α2^ΔEx7^ knockout mice results in cortices with a reduced number of projection neurons in upper and deep layers (Figure 2), therefore explaining the microcephaly noted in newborn mice (Avila et al., 2014).

### GlyR α2 defects lead to hyperexcitation and susceptibility to seizures

Morelli et al. (2017) extended this study by investigating the effect of GlyR α2 ablation on the morphological and functional characteristics of deep-layer neurons of the cerebral cortex. Upon examination of microcephalic GlyR α2^ΔEx7^ knockout mice cortices, a significant reduction in interneurons, as well as upper and sub-cerebral layer projection neurons were recorded when compared to wild-type littermates (Morelli et al., 2017). In addition to a reduction in numbers, morphological defects were seen in the projection neurons and interneurons that populate layer V of the somatosensory cortex. Projection neurons and interneurons of GlyR α2^ΔEx7^ knockout mice cortices were characterized by greater dendritic lengths, as well as increased branching points and dendritic spine numbers. Furthermore, analysis of dendritic shafts and spines found an increased abundance of postsynaptic density 95-positive boutons, suggesting a greater density of excitatory inputs upon projection neurons and interneurons in layer V of GlyR α2^ΔEx7^ knockout somatosensory cortices (Morelli et al., 2017). Subsequent electrophysiological recordings of neonatal layer V projection neurons and interneurons within GlyR α2^ΔEx7^ cortices established a clear shift toward excitation compared to wild-type controls (Morelli et al., 2017). This was evident by an increased frequency of excitatory postsynaptic currents (EPSCs) and a concurrent decreased frequency of inhibitory postsynaptic currents (IPSCs; Morelli et al., 2017). This purported imbalance between excitatory and inhibitory synaptic connections within layer V of the somatosensory cortices was further examined by comparing the behavior of adult GlyR α2^ΔEx7^ knockout and wild-type littermates upon infusion with the chemoconvulsant pentylenetetrazol (Morelli et al., 2017). GlyR α2^ΔEx7^ knockout mice exhibited a significantly lower seizure threshold for the onset of mild and severe behaviors compared to wild-type littermates (Morelli et al., 2017). Taken together, these results suggest that GlyR α2^ΔEx7^ knockout mice have a greater susceptibility to epileptic seizures due to the formation of aberrant somatosensory cortical circuits that are prone to overexcitation.

### GlyR α2 defects in behavior, learning, and memory

Pilorge et al. (2016) also subjected GlyR α2^ΔEx7^ knockout mice to a battery of behavioral tests for motor incoordination, anxiety, repetitive behaviors, and impairments in social interactions. However, GlyR α2^ΔEx7^ knockout mice were indistinguishable from wild-type littermates with regard to these behaviors (Pilorge et al., 2016). Although GlyR α2^ΔEx7^ knockout mice did not display defects in spatial memory as assessed by the novel location recognition task and Morris water maze, they did demonstrate impaired learning and memory in the novel object recognition task (Pilorge et al., 2016). Moreover, prefrontal cortex slices from GlyR α2^ΔEx7^ knockout mice and littermates were subjected to high-frequency stimulation to induce plasticity, with the former displaying impaired long-term potentiation (Pilorge et al., 2016). In subsequent studies, Molchanova et al. (2017) and Comhair et al. (2018) sought to investigate the role of GlyR α2 in striatal medium spiny neuron (MSN) development and function. Firstly, in agreement with previous studies (Jonsson et al., 2012; McCracken et al., 2017), Molchanova et al. (2017) confirmed the expression of GlyR α2 by MSNs of the adult dorsal striatum via immunolabeling. The GlyR α2 subunit was the most abundantly expressed of the four GlyR α subunits, with both MSN populations of the dorsal striatum expressing similar levels (Molchanova et al., 2017). Inhibition of GABA_A_ receptors via the application of gabazine to striatal slices completely abolished IPSCs, suggesting that GlyRs are absent from synaptic sites and might instead be tonically-activated extrasynaptic receptors (Molchanova et al., 2017). Indeed, the reversal potential of glycinergic currents in MSNs was determined to be more positive than the resting membrane potential and the cells treated with strychnine were more hyperpolarized compared to those under control conditions (Molchanova et al., 2017). Therefore, these tonically-active GlyRs exert a depolarizing action upon MSNs at rest and affect the offset of evoked action potential firing (Molchanova et al., 2017). As such, MSNs treated with strychnine or those from GlyR α2^ΔEx7^ knockouts were less excitable and fired fewer action potentials due to their more hyperpolarized resting membrane potential (Molchanova et al., 2017). To assess the potential behavioral consequences of less excitable striatal MSNs, GlyR α2^ΔEx7^ knockouts and wild-type littermates were subjected to a battery of behavioral protocols (Molchanova et al., 2017). In line with previous studies (Young-Pearse et al., 2006; Pilorge et al., 2016; Lin et al., 2017), GlyR α2^ΔEx7^ knockout mice showed no discernible difference in basic locomotion, habituation, and level of anxiety when compared to wild-type littermates (Molchanova et al., 2017). However, GlyR α2^ΔEx7^ knockout mice displayed impaired motor memory consolidation during the rotarod and single-pellet reaching tests (Molchanova et al., 2017).

Comhair et al. (2018) extended these studies by examining the function of GlyR α2 in neonatal MSNs. Curiously, in contrast to previous studies on adult striatal MSNs (Molchanova et al., 2017), in neonatal MSNs no change in holding current was observed upon strychnine application nor were synaptic currents recorded when glutamate and GABA receptors were pharmacologically blocked (Comhair et al., 2018). This suggested that neither tonic nor phasic glycinergic signaling is present in neonatal dorsal MSNs (Comhair et al., 2018). However, despite this apparent lack of tonic or phasic signaling, distinct differences were observed in the characteristics of spontaneous and evoked action potentials between GlyR α2^ΔEx7^ knockout and wild-type neonatal striata (Comhair et al., 2018). The frequency of action potential firing and the threshold at which action potentials began to accommodate were significantly reduced in GlyR α2^ΔEx7^ knockout striata compared to controls (Comhair et al., 2018). Additionally, individual action potential amplitudes were lower, and their duration was longer in GlyR α2^ΔEx7^ knockout striata (Comhair et al., 2018). These results were replicated upon strychnine application to wild-type striata, validating the involvement of GlyR α2 in the spontaneous activity of neonatal MSNs (Comhair et al., 2018). Functional analysis of the glutaminergic innervation of neonatal MSNs found that the frequency of miniature EPSCs was reduced in GlyR α2^ΔEx7^ knockout striata and this impairment persisted into adulthood (Comhair et al., 2018). Unlike the aberrant dendritic morphology which was observed in projection neurons and interneurons of the cerebral cortex in GlyR α2^ΔEx7^ knockout mice (Morelli et al., 2017), striatal MSN dendritic tree morphology and glutamatergic synapse abundance remained unaltered between the GlyR α2^ΔEx7^ knockout and controls (Comhair et al., 2018). However, compared to wild-type striata, a reduction in the AMPA/NMDA ratio was observed in GlyR α2^ΔEx7^ knockout mice, indicative of a greater number of silent synapses and therefore a deficit in synapse maturation (Comhair et al., 2018). To investigate whether this immature glutamatergic input onto striatal MSNs would result in impaired motor performance, neonatal GlyR α2^ΔEx7^ knockout mice and littermate controls were examined in the righting reflex test (Comhair et al., 2018). Indeed, GlyR α2^ΔEx7^ knockout neonates were significantly slower to right to all four paws starting from P6 compared to their wild-type counterparts (Comhair et al., 2018).

### GlyR α2 defects increase dopaminergic signaling and enhance reward-motivated behaviors

The dorsal striatum also mediates reward-motivated behaviors (Balleine et al., 2007). Dopaminergic input from the midbrain to striatal MSNs of the direct pathway (which express dopamine D1 receptors) causes GABAergic inhibition of the internal globus pallidus and substantia nigra pars reticulata nuclei (Yager et al., 2015). The resulting excitation of the thalamus provides a ‘go’ signal to commence behaviors (Yager et al., 2015). Devoght et al. (2023) sought to examine the effect of GlyR α2^ΔEx7^ knockout on dopamine-mediated striatal activity and function in adult mice. GlyR α2 expressed by D1-MSNs moderates dopamine-mediated activity, acting to shunt striatal cell depolarization as the membrane potential exceeds the Cl^−^ equilibrium potential (-54 mV) (Devoght et al., 2023). Devoght et al. (2023) therefore hypothesized that depletion of GlyR α2 would allow a larger increase in striatal projection neuron (SPN) activity in response to dopamine. Indeed, optogenetically induced dopamine release on SPNs in GlyR α2^ΔEx7^ knockout brain slices revealed a greater increase in the dopamine-mediated activity of D1-MSNs, compared to wild-type controls (Devoght et al., 2023). Consistent with this increased dopamine release, GlyR α2^ΔEx7^ mice demonstrated an increase in novelty-induced locomotion in the open field test, with increased time spent in the center of the arena (Devoght et al., 2023). An increased locomotor response to D-amphetamine (but not cocaine) was also observed in GlyR α2^ΔEx7^ knockout mice. GlyR α2^ΔEx7^ knockout and wild-type mice were also subjected to an appetitive conditioning task, where difference only became apparent during highly-demanding motivational reward schedules, indicative of enhanced motivated behavior in GlyR α2^ΔEx7^ knockout mice (Devoght et al., 2023).

## GlyR α2 variants in human neurodevelopmental disorders

In addition to the studies documented above for GlyR α2 subunit knockout mice, investigation of a potential role for GlyR α2 dysfunction in human neurological disorders was first prompted after several large-scale sequencing projects documented rare *GLRA2* missense variants in isolated cases of autism spectrum disorder (ASD; Pinto et al., 2010; Piton et al., 2011; Iossifov et al., 2014; Pilorge et al., 2016; Supplementary Table S1). Additional clinical symptoms were reported in some individuals, including delay/loss of acquired language and seizures (Piton et al., 2011; Pilorge et al., 2016). The first structure–function study on GlyR α2 variants reported a microdeletion (*GLRA2*^ΔEx8–9^) and two *de novo* missense mutations, GlyR α2^N109S^ and α2^R126Q^, found in the hemizygous (XY) state in males (Pilorge et al., 2016). All three of these variants resulted in a total or partial *loss-of-function* (Pilorge et al., 2016). Microdeletion *GLRA2*^ΔEx8–9^ was reported in a male with ASD, motor incoordination, language delay and bilateral myopia, which he had inherited from his healthy mother (Pinto et al., 2010; Pilorge et al., 2016). This microdeletion results in the truncation of GlyR α2 in the ICD, with loss of TM4. Although the resulting transcript appears to escape nonsense-mediated RNA decay, the truncated GlyR α2 subunit is not expressed at the cell surface (Pilorge et al., 2016). Given that *GLRA2* escapes X-inactivation in most tissues including the brain (Cotton et al., 2015), the mother of the proband likely remains unaffected by this microdeletion as the normal allele can compensate for the microdeletion. By contrast, GlyR α2^N109S^ and α2^R126Q^ missense variants cause reduced whole-cell and cell-surface expression and loss of glycine sensitivity (Pilorge et al., 2016). Consistent with this finding, molecular modeling predicted that the α2^R126Q^ substitution abolishes critical hydrogen bonds within the glycine binding site (Pilorge et al., 2016). A third variant, GlyR α2^R323L^, was identified in the heterozygous state in a female ASD proband, inherited from her healthy mother (Piton et al., 2011). Other clinical features included a loss of acquired words, seizures, mild motor development delay, macrocephaly and hypothyroidism (Supplementary Table S1). This case was of interest, given that microcephaly was observed in newborn *Glra2* knockout mice (Avila et al., 2014). Could a *gain-of-function* mutation explain the macrocephaly? Curiously, detailed functional analysis revealed that GlyR α2^R323L^ does indeed result in a *gain-of-function* (Zhang et al., 2017) due to slower synaptic decay times, longer durations of active periods and an increase in single-channel conductance for both homomeric α2^R323L^ and heteromeric α2^R323L^β receptors (Zhang et al., 2017).

More recently, Chen et al. (2022) published a comprehensive functional analysis of four missense mutations GlyR α2^V-22L^, GlyR α2^N38K^, GlyR α2^K213E^ and GlyR α2^T269M^ (Supplementary Table S1). GlyR α2^V-22L^ is located in the N-terminal signal peptide, and was originally in a female ASD proband with a verbal and non-verbal IQ of 63 and 103 (Supplementary Table S1). This was initially dismissed, since a variant in the signal peptide is not normally expected to disrupt GlyR α2 function, as it is cleaved during receptor subunit maturation and assembly. However, Chen et al. (2022) found that bioinformatic algorithms predicted that GlyR α2^V-22L^ alters the signal peptide cleavage site, resulting in the retention of an additional five amino acids at the GlyR α2 subunit N-terminus. Biochemical analysis found that while whole-cell expression of GlyR α2^V-22L^ was unaltered, cell-surface expression was significantly reduced, consistent with a partial *loss-of-function*. GlyR α2^N38K^ was a *de novo* variant found in a male assigned as a ‘designated unaffected sibling’ of an ASD case (Supplementary Table S1; Krumm et al., 2015). However, molecular modeling revealed that the GlyR α2^N38K^ variant introduces a clash with the glycan attached to residue N45, possibly impeding N-linked glycosylation of GlyR α2 (Chen et al., 2022). Subsequent biochemical assays demonstrated that GlyR α2^N38K^ exhibits significantly reduced whole-cell and cell-surface expression (Chen et al., 2022). In line with a diminished cell-surface expression, GlyR α2^N38K^ exhibited a reduced I_max_ when expressed in HEK293 cells, as well as smaller IPSC amplitudes in artificial synapses (Chen et al., 2022). This partial *loss-of-function* is consistent with previous studies of artificial N-glycosylation site mutants in GlyR α1, which impair receptor homo-oligomerization and transit through the ER-Golgi complex toward the cell membrane (Griffon et al., 1999; Schaefer et al., 2015, 2018). A male NDD case featuring refractory epilepsy, microcephaly, and severe developmental delay was identified by Chen et al. (2022) via diagnostic exome sequencing. GlyR α2^K213E^ affects a highly conserved residue within the ECD, which is situated among important agonist-binding residues (Y209, T211 and F214; Yu et al., 2021; Chen et al., 2022). Molecular modeling demonstrated that GlyR α2^K213E^ resulted in a clash with H208 in the closed state, while forming additional contacts with Y209 in the open state, suggesting that the ligand-bound open state might be favored (Chen et al., 2022). Although biochemical assays revealed a slight reduction in cell-surface expression, IPSCs generated by GlyR α2^K213E^ in artificial synapses had significantly larger amplitudes, faster rise times and slower decay times, suggesting a *gain-of-function* (Chen et al., 2022).

The most common missense variant is GlyR α2^T269M^, which affects a highly conserved residue within the pore-forming TM2 domain (Chen et al., 2022; Marcogliese et al., 2022). Initially identified in a female proband by a study that reported *de novo* variants in individuals with developmental disorders (Deciphering Developmental Disorders Study, 2017), this missense mutation has since been described in an additional six female NDD probands, all of whom have a *de novo* mode of inheritance (Marcogliese et al., 2022). These six cases have a diverse array of clinical features, including DD/ID, hypotonia/incoordination, ASD, inattention/hyperactivity, sleep disturbance, microcephaly, epilepsy, as well as various ocular defects (Supplementary Table S1). Using molecular dynamics simulations, Chen et al. (2022) revealed that GlyR α2^T269M^ channels are predicted to allow an increased occupancy of water and Cl^−^ within the channel pore (Chen et al., 2022). Consistent with these simulations, GlyR α2^T269M^ homomers exhibit a significant leak current as revealed by blockage with picrotoxin, and a reduced glycine EC_50_ value (Chen et al., 2022). Balancing these findings with a reduced cell-surface expression and whole-cell I_max_, Chen et al. (2022) categorized GlyR α2^T269M^ overall as an *alteration-of-function* variant (Supplementary Table S1). Interestingly, missense mutations that result in spontaneous leak currents have also been previously reported in GlyR α1 in startle disease/hyperekplexia (e.g., GlyR α1^Q226E^, GlyR α1^V280M^, and GlyR α1^R414H^). These mutations act in a similar manner, prolonging the decay of inhibitory postsynaptic currents (IPSCs) in artificial synapses and inducing spontaneous GlyR activation (Chung et al., 2010; Bode et al., 2013; Zhang et al., 2016).

**Figure 3:**
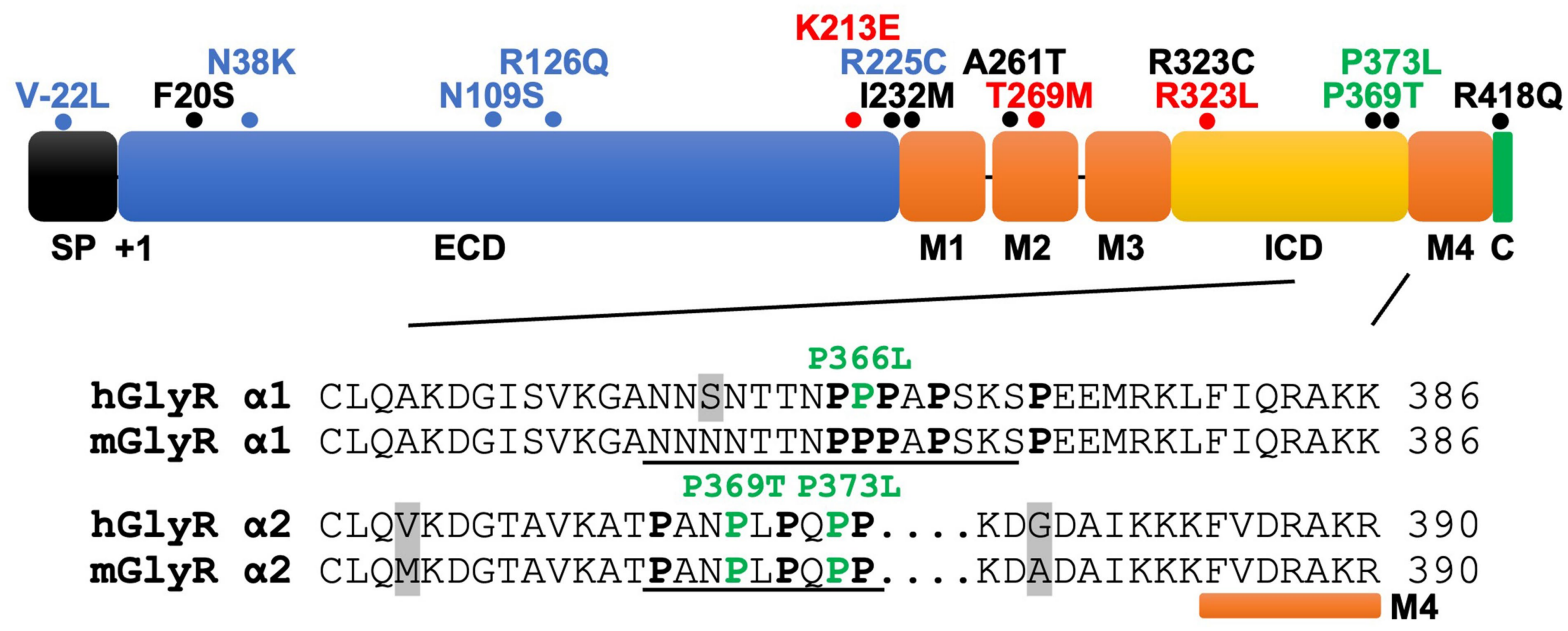
GlyR α2 subunit NDD mutations fall into key functional domains. Domain structure of the GlyR α2 subunit, including an N-terminal signal peptide (SP), extracellular domain (ECD), four membrane-spanning domains (M1-M4), the intracellular domain (ICD) and the extracellular C-terminus (C). For GlyR α2 missense variants (mature protein numbering), blue = *loss-of-function*; red = *gain-of-function; green = potential altered PPIs; black = unknown. Inset*: Alignment of mouse and human GlyR α1-α2 subunit ICD sequences prior to M4. The GlyR α2 variants p.P369T/p.P373L form part of a tandem PXXP motif (underlined) and are predicted to disrupt interactions with as yet uncharacterized GlyR α2 interacting proteins containing a SH3 domain. While syndapin I is known to bind to a proline-rich sequence in GlyR α1 (underlined) and this interaction is disrupted by the GlyR α1^P366L^ startle disease missense variant, there is currently no biochemical evidence that syndapin I or the RhoGEF collybistin binds to the GlyR α2 subunit. Proline residues are highlighted in bold type and grey shading indicates differences between mouse/human sequences.

Lastly, Marcogliese et al. (2022) also reported GlyR α2 missense variants in 13 unrelated NDD probands with heterogeneous clinical features, including ASD, developmental and cognitive delay of varying severity, motor incoordination, epilepsy, sleep disturbances, microcephaly, ocular manifestations, and dysmorphic craniofacial features (Supplementary Table S1). As previously mentioned, six of these probands are females who carry the recurrent *de novo* GlyR α2^T269M^ variant (Marcogliese et al., 2022). Using a novel *Drosophila*-based functional system, Marcogliese et al. (2022) classified GlyR α2^T269M^ as a *gain-of-function* allele based on overexpression of human GlyR α2^T269M^ in presynaptic photoreceptors and postsynaptic neurons, reporting a significant increase in amplitudes of “OFF” transients for the GlyR α2^T269M^ transgenic line. Using this system, they also classified GlyR α2^R225C^ as a *loss-of-function* allele (Marcogliese et al., 2022). However, this system has substantial limitations for the study of GlyR α2 subunit mutants, since glycinergic neurons in *Drosophila* seem to be limited to small ventral lateral neurons (sLNvs) involved in circadian behavior (Frenkel et al., 2017). Thus, it is unclear how glycine is released onto exogenous GlyRs expressed in photoreceptors. In addition, this system does not measure defects in cell-surface trafficking or spontaneous opening of GlyRs. Other missense variants identified by Marcogliese et al. (2022) including p.F20S, p.I232M, p.A261T, p.P369T, p.P373L, p.R418Q. Another recently reported missense variant in the ICD (p.R323C; Mir et al., 2023) affects the same amino acid as the known *gain-of-function* mutant GlyR α2^R323L^ (Figure 3; Supplementary Table S1).

### Are defective GlyR α2-accessory protein interactions a hidden cause of NDDs?

Sequence alignments reveal that p.P369T and p.P373L are located in the GlyR α2 TM3-TM4 ICD in a conserved polyproline helix type II (PPII), a helical secondary structure known for mediating interactions with *src* homology 3 (SH3) domain-containing proteins (Langlhofer and Villmann, 2016; Figure 3). This is of significant interest, since proline-rich stretches of the GlyR α1 and β subunit TM3-TM4 ICDs are involved in interactions with several accessory proteins (Del Pino et al., 2014; Langlhofer et al., 2020; Breitinger et al., 2021). A proline-rich motif in the GlyR β subunit mediates interactions with the SH3 domain of syndapin I, a neuronally expressed member of the Fes/CIP4 homology Bin-Amphiphysin-Rvs (F-BAR) family of lipid binding and remodeling proteins (Del Pino et al., 2014). Syndapin I has been linked to a physiological role in activity-dependent bulk endocytosis (Quan and Robinson, 2013). Using a syndapin I knockout mouse model, it was found that syndapin I regulates GlyR β cluster size, density and mobility, as well as internalization via GlyR β decoupling from synaptic gephyrin scaffolds (Troger et al., 2022).

Syndapin I also associates with the GlyR α1 subunit ICD, albeit with substantially lower affinity (Langlhofer et al., 2020). Interestingly, a missense variant associated with startle disease in the PPII helix, GlyR α1^P366L^ subunit (Figure 3) has been proposed to disrupt interactions with syndapin I. GlyR α1^P366L^ did not affect whole-cell or cell-surface receptor expression (Langlhofer et al., 2020). However, GlyR α1^P366L^ and α1^P366L^β receptors had a reduced I_max_, rapid desensitization kinetics, and exhibited spontaneous channel openings in the absence of glycine as well as a reduction in single-channel conductance (Langlhofer et al., 2020). *In vitro* analysis using peptide microarrays and tandem MS-based analysis methods suggested that binding between the PPII helix of the GlyR α1 subunit and the SH3 domain of syndapin I was disrupted upon introduction of the GlyR α1^P366L^ mutation. However, this was not reflected within an *ex vivo* context, as incubation of whole-brain lysates from adult mice with resin-bound GlyR α1 and GlyR α1^P366L^ peptides revealed a clear enrichment of syndapin I in the interactomes of both wild-type and mutant subunits (Langlhofer et al., 2020). Finally, Langlhofer et al. (2020) examined the neuronal distribution of syndapin I in primary hippocampal cultures virally infected with either GlyR α1 or GlyR α1^P366L^ constructs. When compared to neurons expressing wild-type GlyR α1, those transfected with GlyR α1^P366L^ preferentially accumulated syndapin I in the cell soma rather than neurites (Langlhofer et al., 2020).

Subsequent to these findings, Breitinger et al. (2021) investigated whether GlyR α1^P366L^ also interferes with binding to the RhoGEF collybistin, another SH3 domain-containing protein crucial for gephyrin clustering at inhibitory synapses (Kins et al., 2000; Harvey K. et al., 2004). Unexpectedly, while GST-pulldown assays did reveal an interaction between the GlyR α1 TM3-TM4 ICD and collybistin, the site of this interaction was localized to the pleckstrin (PH) domain of collybistin, rather than the SH3 domain (Breitinger et al., 2021). A comparison of the co-localization of wild-type GlyR α1 and GlyR α1^P366L^ with collybistin in cultured hippocampal neurons demonstrated that p.P366L appeared to weaken the colocalization of GlyR α1 with collybistin in the cell soma, but not dendrites (Breitinger et al., 2021). In accordance with these findings, the novel NDD-associated variants in the proline-rich region of the GlyR α2 TM3-TM4 ICD (p.P369T and p.P373L) (Figure 3) may similarly disrupt interactions with uncharacterized SH3-domain containing proteins that interact with GlyR α2.

## Conclusions and future perspectives

**Figure 4:**
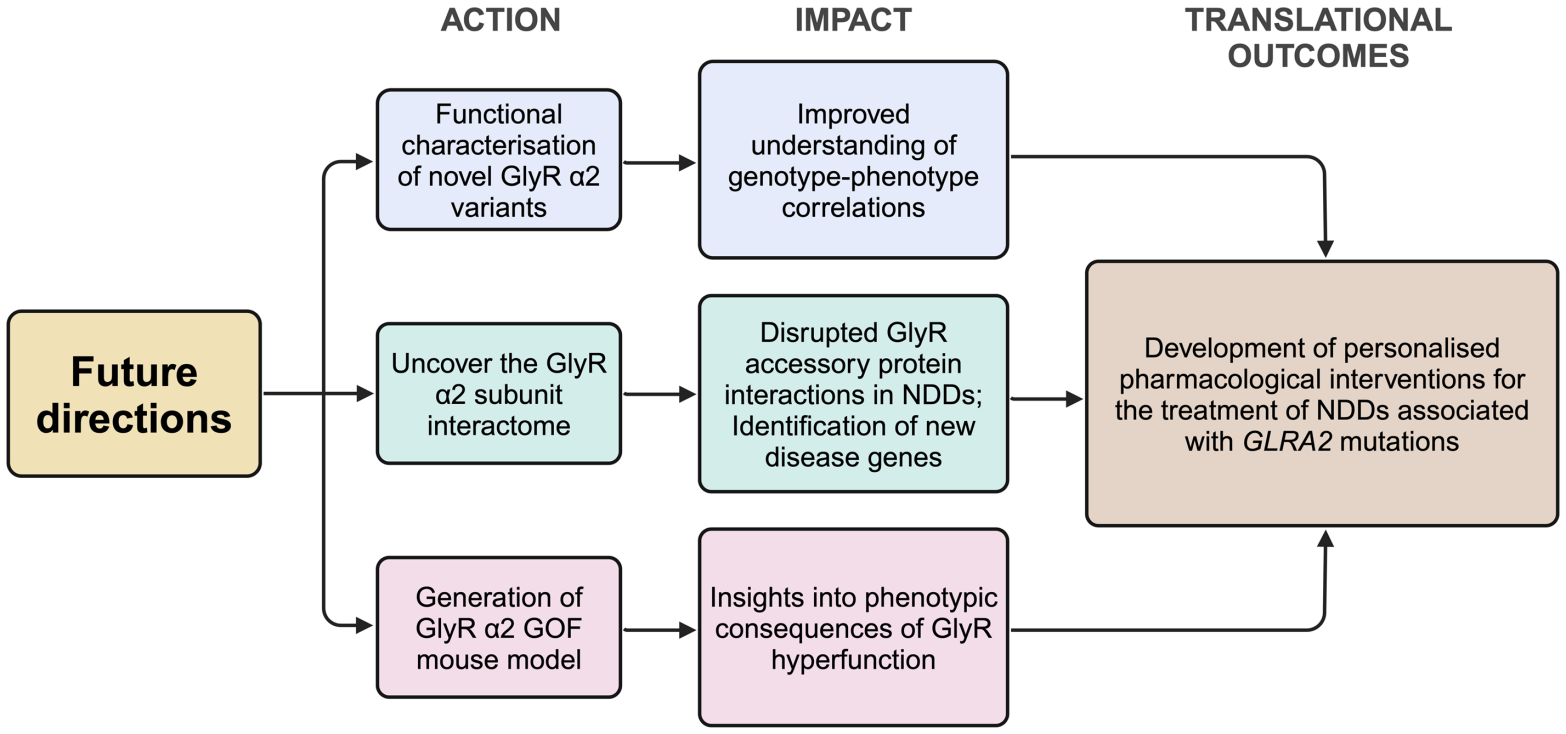
Proposed future directions for the study of the role of the GlyR α2 subunit in health and disease. GlyR, glycine receptor; KI, knock-in; NDD, neurodevelopmental disorder. Created in https://BioRender.com.

The generation and analysis of *Glra2* knockout mouse models has afforded valuable insights into the role of this GlyR subtype in health and disease (Figure 1), revealing roles in cortical progenitor homeostasis and interneuron migration (Avila et al., 2013; Avila et al., 2014), object recognition memory and motor memory consolidation (Pilorge et al., 2016; Molchanova et al., 2017), increased susceptibility to seizures (Morelli et al., 2017), defects in righting reflexes (Comhair et al., 2018), increased novelty-induced locomotion in the open field test and increased performance in motivational reward tasks (Devoght et al., 2023). GlyR α2^ΔEx7^ knockout mice have also implicated GlyR α2 in ethanol-related behaviors, including binge-like drinking (San Martin et al., 2020). Since the initial discovery of a GlyR α2 microdeletion and missense variants in isolated cases of autism spectrum disorder with accompanying language defects and/or seizures (Pinto et al., 2010; Piton et al., 2011; Iossifov et al., 2014; Pilorge et al., 2016), GlyR α2 missense variants have been identified in a spectrum of cases encompassing autism spectrum disorder, intellectual disability and neurodevelopmental disorders (Deciphering Developmental Disorders Study, 2017; Chen et al., 2022; Marcogliese et al., 2022; Mir et al., 2023). Importantly, functional studies have discovered that *GLRA2* variants can result in a loss, gain or alteration of GlyR function (Supplementary Table S1; Pilorge et al., 2016; Zhang et al., 2017; Chen et al., 2022; Marcogliese et al., 2022), perhaps explaining the broad range of clinical phenotypes observed. Further GlyR α2 genetic variants are expected to be identified as *GLRA2* becomes integrated into screening panels for a range of neurological disorders. In this context, there are several avenues of investigation that will enhance our understanding of the role of GlyR α2 in health and disease, and provide potential routes to personalized pharmacotherapies (Figure 4). However, despite these successes – it is worth noting the shortcomings of current functional studies, GlyR α2 mouse models and key knowledge gaps.

Firstly, while functional studies have revealed that GlyR α2 missense variants can result in loss, gain, or altered function, there is no clear correlation between the nature of the GlyR dysfunction and resulting clinical manifestations, severity, or disease prognosis. There are several potential confounding factors that may affect this analysis. For example, sex-specific differences have been reported in the expression of GlyR α2 subunit transcripts in the cortex, striatum, hypothalamus and brainstem (Ceder et al., 2024) which may influence disease severity in males versus females. In addition, NDDs often display a notable male bias in prevalence (Mendes et al., 2025). This ‘female protective effect’ suggests that females may require a higher genetic burden to manifest symptoms similar to those in males. This is relevant in for GlyR α2, since the corresponding gene is X-linked, meaning that defects in a single allele in males (XY) are highly likely to be pathogenic. However, there are also documented cases where *de novo* or inherited variants affecting single GlyR α2 alleles in females are associated with disease (e.g., p.V-22 L, p.F20S, p.I232M, p.T269M, p.R323C, Supplementary Table S1). In this context, it is important to note that the GlyR α2 gene escapes X-inactivation in humans in the vast majority of tissues including the brain (Cotton et al., 2015). This means that both wild-type and defective alleles will be co-expressed, and since heteromeric α2β GlyRs can contain four α2 subunits, most receptors will contain one or more defective α2 subunits. Lastly, seven out of the sixteen known GlyR α2 variants remain functionally uncharacterized (Figure 3; Supplementary Table S1). We anticipate that further exploration of sex-specific differences in GlyR α2 knockout mouse models and further experimental validation of GlyR α2 missense variants in native GlyR α2β combinations, or in the artificial synapse system (Zhang Y. et al., 2015; Zhang et al., 2017; Chen et al., 2022), will substantially improve our understanding of genotype–phenotype correlations for GlyR α2.

Secondly, the discovery GlyR α2 missense variants affecting residues in intracellular polyproline motifs (Marcogliese et al., 2022) has also revealed a major knowledge gap – we currently do not know the identity of any SH3-domain containing proteins that interact with the GlyR α2 subunit. To date, only one previous study has sought to identify GlyR α2 interactors, using pulldown essays on rat brain extracts using a GST fusion protein containing the GlyR α2 subunit TM3-TM4 loop as bait (Bluem et al., 2007). This experimental design resulted in an enigmatic assortment of proteins, including components of the translational machinery [eukaryotic elongation factor 1α (eEF1A), p70 ribosomal protein S6 kinase (rS6 kinase) and ribosomal protein S6 (rpS6); Bluem et al., 2007]. However, follow-up assays did not reveal whether these proposed interactors modulated GlyR α2 expression and/or function (Bluem et al., 2007), casting doubt as to whether these proteins represent genuine constituents of the GlyR α2 interactome *in vivo*. The GlyR α2 subunit TM3-TM4 loop possesses a proline-rich SH3 domain-binding motif similar to those found in GlyR α1 and β subunits, which have been found to bind the F-BAR and SH3 domain-containing protein syndapin I (Figure 3). For GlyR α1 and β subunits, interactions with syndapin I could be disrupted by mutations within intracellular polyproline motifs, such as GlyR α1^P366L^ and GlyR β^P438A/P441A^ (Del Pino et al., 2014; Langlhofer et al., 2020). However, there is currently no evidence that syndapin I or another key SH3-domain protein found at inhibitory synapses – the RhoGEF collybistin (Kins et al., 2000; Harvey K. et al., 2004) – binds to GlyR α2 *in vitro* or *in vivo*. While one study has suggested that GlyR α1 binds directly to collybistin, this interaction appeared to be mediated by the collybistin pleckstrin homology (PH) domain, rather than the SH3 domain (Breitinger et al., 2021). It is therefore imperative that the GlyR α2 interactome is re-investigated using modern proteomic methods as a matter of priority. New high-resolution methods, incorporating GlyR α2 knockout mouse tissue as a negative control, may reveal new components of a signaling complex associated with cortical development and function. One exciting prospect is that GlyR α2 subunit interactors may themselves be involved in the pathogenesis of NDDs, e.g., reciprocal SH3 domain mutations might disrupt interactions with the GlyR α2 subunit ICD.

Thirdly, it is clear that the generation and analysis of *Glra2* knockout mouse models has facilitated insights into the morphological, physiological and behavioral consequences of ablating GlyR α2 subunit expression and function (Figure 1). However, all existing mouse models (GlyR α2^ΔEx6–7^: Young-Pearse et al., 2006; GlyR α2^ΔEx7^: Avila et al., 2013; GlyR α2^ΔEx2^: Tian et al., 2023) represent *loss-of-function* alleles. To fully understand the consequences of GlyR α2 *gain-of-function* or *alteration-of-function* variants (e.g., GlyR α2^T269M^ or GlyR α2^R323L^; Zhang et al., 2017; Chen et al., 2022) that have the potential to *potentiate* glycinergic signaling, novel first-in-class *gain-of-function* mouse models are required. The GlyR α2^R323L^ variant represents an excellent candidate for a new mouse model of GlyR α2 *hyperfunction*, since this missense variant does not affect cell-surface trafficking, and incorporates into both homomeric GlyR α2 and heteromeric GlyR α2β complexes. GlyR α2^R323L^ also has slower IPSC decay times and longer durations of active periods in artificial synapses due to increased single-channel conductance in the presence of glycine (Zhang et al., 2017). However, the *alteration-of-function* variant GlyR α2^T269M^ is also a compelling candidate – since it is associated with multiple cases of NDDs and spontaneously-opening GlyRs (Chen et al., 2022; Marcogliese et al., 2022). We anticipate that new GlyR α2 *gain-of-function* or *alteration-of-function* mouse lines will represent important new tools for understanding processes underlying brain development, physiology, learning/memory, seizure susceptibility and alcohol-related behaviors.

Finally, the ultimate translational outcome of this research would be personalized pharmacotherapies for individuals with GlyR α2 missense variants. Studies aimed at understanding exactly how disease-associated variants affect GlyR α2 structure and function will lead to insights into how to therapeutically-target defective GlyRs. For instance, GlyR α2 *loss-of-function* variants that affect cell-surface expression could potentially be treated with proteostasis regulators such as suberanilohydroxamic acid, dinoprost and dihydroergocristine, which have been found to restore surface trafficking of GABA_A_R epilepsy mutants (Durisic et al., 2018; Di et al., 2021). Alternatively, drugs from the GlyR allosteric ligands library (GRALL; Cerdan et al., 2020) could be tested for potentiation of wild-type GlyR α2 and partial *loss-of-function* mutants. Certainly, *gain-of-function* variants that potentiate glycinergic signaling may be responsive to pharmacotherapy with GlyR subunit-specific antagonists. Current candidate molecules include cyclothiazide and curcumol, both of which exert a concentration-dependent reduction in glycine-activated currents mediated by homomeric GlyR α2 and heteromeric GlyR α2β channels (Zhang et al., 2008; Wang et al., 2012). However, these and any other potential pharmacotherapies would need detailed testing in pre-clinical models, such as novel GlyR α2 *gain-of-function* mouse models. Certainly, the last ten years have seen an explosion of knowledge in terms of understanding the role of the GlyR α2 subunit in health and disease, and we look forward to seeing what insights the next ten years will bring.

## References

[ref1] Akagi H. Hirai K. Hishinuma F. (1991). Cloning of a glycine receptor subtype expressed in rat brain and spinal cord during a specific period of neuronal development. FEBS Lett. 281, 160–166. doi: 10.1016/0014-5793(91)80383-e, PMID: 1707830

[ref2] Avila A. Vidal P. M. Dear T. N. Harvey R. J. Rigo J. M. Nguyen L. (2013). Glycine receptor α2 subunit activation promotes cortical interneuron migration. Cell Rep. 4, 738–750. doi: 10.1016/j.celrep.2013.07.016, PMID: 23954789 PMC3763372

[ref3] Avila A. Vidal P. M. Tielens S. Morelli G. Laguesse S. Harvey R. J.. (2014). Glycine receptors control the generation of projection neurons in the developing cerebral cortex. Cell Death Differ. 21, 1696–1708. doi: 10.1038/cdd.2014.75, PMID: 24926615 PMC4211368

[ref4] Balleine B. W. Delgado M. R. Hikosaka O. (2007). The role of the dorsal striatum in reward and decision-making. J. Neurosci. 27, 8161–8165. doi: 10.1523/jneurosci.1554-07.2007, PMID: 17670959 PMC6673072

[ref5] Becker C. M. Hoch W. Betz H. (1988). Glycine receptor heterogeneity in rat spinal cord during postnatal development. EMBO J. 7, 3717–3726. doi: 10.1002/j.1460-2075.1988.tb03255.x, PMID: 2850172 PMC454946

[ref6] Blednov Y. A. Benavidez J. M. Black M. Leiter C. R. Osterndorff-Kahanek E. Harris R. A. (2015). Glycine receptors containing α2 or α3 subunits regulate specific ethanol-mediated behaviors. J. Pharmacol. Exp. Ther. 353, 181–191. doi: 10.1124/jpet.114.221895, PMID: 25678534 PMC4366753

[ref7] Bluem R. Schmidt E. Corvey C. Karas M. Schlicksupp A. Kirsch J.. (2007). Components of the translational machinery are associated with juvenile glycine receptors and are redistributed to the cytoskeleton upon aging and synaptic activity. J. Biol. Chem. 282, 37783–37793. doi: 10.1074/jbc.M708301200, PMID: 17965018

[ref8] Bode A. Wood S. E. Mullins J. G. L. Keramidas A. Cushion T. D. Thomas R. H.. (2013). New hyperekplexia mutations provide insight into glycine receptor assembly, trafficking, and activation mechanisms. J. Biol. Chem. 288, 33745–33759. doi: 10.1074/jbc.M113.509240, PMID: 24108130 PMC3837119

[ref9] Bormann J. Rundstrom N. Betz H. Langosch D. (1993). Residues within transmembrane segment M2 determine chloride conductance of glycine receptor homo- and hetero-oligomers. EMBO J. 12, 3729–3737. doi: 10.1002/j.1460-2075.1993.tb06050.x, PMID: 8404844 PMC413654

[ref10] Breitinger H. G. Becker C. M. (2002). The inhibitory glycine receptor-simple views of a complicated channel. Chembiochem 3, 1042–1052. doi: 10.1002/1439-7633(20021104)3:11<1042::AID-CBIC1042>3.0.CO;2-7, PMID: 12404628

[ref11] Breitinger U. Weinlander K. Pechmann Y. Langlhofer G. Enz R. Becker C. M.. (2021). A proline-rich motif in the large intracellular loop of the glycine receptor α1 subunit interacts with the pleckstrin homology domain of collybistin. J. Adv. Res. 29, 95–106. doi: 10.1016/j.jare.2020.09.009, PMID: 33842008 PMC8020344

[ref12] Buerbank S. Becker K. Becker C. M. Brandt N. Engel J. Knipper M.. (2011). Developmental regulation of glycine receptors at efferent synapses of the murine cochlea. Histochem. Cell Biol. 136, 387–398. doi: 10.1007/s00418-011-0855-6, PMID: 21850450

[ref13] Carta E. Chung S. K. James V. M. Robinson A. Gill J. L. Remy N.. (2012). Mutations in the GlyT2 gene (*SLC6A5*) are a second major cause of startle disease. J. Biol. Chem. 287, 28975–28985. doi: 10.1074/jbc.M112.372094, PMID: 22700964 PMC3436555

[ref14] Ceder M. M. Magnusson K. A. Weman H. M. Henriksson K. Andréasson L. Lindström T.. (2024). The mRNA expression profile of glycine receptor subunits α1, α2, α4 and β in female and male mice. Mol. Cell. Neurosci. 131:103976. doi: 10.1016/j.mcn.2024.103976, PMID: 39580061

[ref15] Cerdan A. H. Sisquellas M. Pereira G. Barreto Gomes D. E. Changeux J. P. Cecchini M. (2020). The glycine receptor allosteric ligands library (GRALL). Bioinformatics 36, 3379–3384. doi: 10.1093/bioinformatics/btaa170, PMID: 32163115 PMC7267813

[ref16] Chen X. Wilson K. A. Schaefer N. De Hayr L. Windsor M. Scalais E.. (2022). Loss, gain and altered function of GlyR α2 subunit mutations in neurodevelopmental disorders. Front. Mol. Neurosci. 15:886729. doi: 10.3389/fnmol.2022.886729, PMID: 35571374 PMC9103196

[ref17] Chung S. K. Vanbellinghen J. F. Mullins J. G. Robinson A. Hantke J. Hammond C. L.. (2010). Pathophysiological mechanisms of dominant and recessive *GLRA1* mutations in hyperekplexia. J. Neurosci. 30, 9612–9620. doi: 10.1523/jneurosci.1763-10.2010, PMID: 20631190 PMC6632444

[ref18] Comhair J. Devoght J. Morelli G. Harvey R. J. Briz V. Borrie S. C.. (2018). Alpha2-containing glycine receptors promote neonatal spontaneous activity of striatal medium spiny neurons and support maturation of glutamatergic inputs. Front. Mol. Neurosci. 11:380. doi: 10.3389/fnmol.2018.00380, PMID: 30374290 PMC6196267

[ref19] Cotton A. M. Price E. M. Jones M. J. Balaton B. P. Kobor M. S. Brown C. J. (2015). Landscape of DNA methylation on the X chromosome reflects CpG density, functional chromatin state and X-chromosome inactivation. Hum. Mol. Genet. 24, 1528–1539. doi: 10.1093/hmg/ddu564, PMID: 25381334 PMC4381753

[ref20] Darwish M. Hattori S. Nishizono H. Miyakawa T. Yachie N. Takao K. (2023). Comprehensive behavioral analyses of mice with a glycine receptor α4 deficiency. Mol. Brain 16:44. doi: 10.1186/s13041-023-01033-x, PMID: 37217969 PMC10201759

[ref21] Deciphering Developmental Disorders Study (2017). Prevalence and architecture of *de novo* mutations in developmental disorders. Nature 542, 433–438. doi: 10.1038/nature21062, PMID: 28135719 PMC6016744

[ref22] Del Pino I. Koch D. Schemm R. Qualmann B. Betz H. Paarmann I. (2014). Proteomic analysis of glycine receptor β subunit (GlyR β)-interacting proteins: evidence for syndapin I regulating synaptic glycine receptors. J. Biol. Chem. 289, 11396–11409. doi: 10.1074/jbc.M113.504860, PMID: 24509844 PMC4036276

[ref23] Devoght J. Comhair J. Morelli G. Rigo J.-M. D'Hooge R. Touma C.. (2023). Dopamine-mediated striatal activity and function is enhanced in GlyR α2 knockout animals. iScience 26:107400. doi: 10.1016/j.isci.2023.107400, PMID: 37554441 PMC10404725

[ref24] Di X. J. Wang Y. J. Cotter E. Wang M. Whittsette A. L. Han D. Y.. (2021). Proteostasis regulators restore function of epilepsy-associated GABA_A_ receptors. Cell Chem. Biol. 28:e47, 46–59.e7. doi: 10.1016/j.chembiol.2020.08.012, PMID: 32888501 PMC7855620

[ref25] Dlugaiczyk J. Hecker D. Neubert C. Buerbank S. Campanelli D. Becker C. M.. (2016). Loss of glycine receptors containing the α3 subunit compromises auditory nerve activity, but not outer hair cell function. Hear. Res. 337, 25–34. doi: 10.1016/j.heares.2016.05.004, PMID: 27208792

[ref26] Drehmann P. Milanos S. Schaefer N. Kasaragod V. B. Herterich S. Holzbach-Eberle U.. (2023). Dual role of dysfunctional Asc-1 transporter in distinct human pathologies, human startle disease, and developmental delay. eNeuro 10:ENEURO.0263-23.2023. doi: 10.1523/ENEURO.0263-23.2023, PMID: 37903619 PMC10668224

[ref27] Du J. Lu W. Wu S. Cheng Y. Gouaux E. (2015). Glycine receptor mechanism elucidated by electron cryo-microscopy. Nature 526, 224–229. doi: 10.1038/nature14853, PMID: 26344198 PMC4659708

[ref28] Durisic N. Keramidas A. Dixon C. L. Lynch J. W. (2018). SAHA (Vorinostat) corrects inhibitory synaptic deficits caused by missense epilepsy mutations to the GABA_A_ receptor γ2 subunit. Front. Mol. Neurosci. 11:89. doi: 10.3389/fnmol.2018.00089, PMID: 29628874 PMC5876238

[ref29] Flint A. C. Liu X. L. Kriegstein A. R. (1998). Nonsynaptic glycine receptor activation during early neocortical development. Neuron 20, 43–53. doi: 10.1016/S0896-6273(00)80433-X, PMID: 9459441

[ref30] Frenkel L. Muraro N. I. Beltrán González A. N. Marcora M. S. Bernabó G. Hermann-Luibl C.. (2017). Organization of circadian behavior relies on glycinergic transmission. Cell Rep. 19, 72–85. doi: 10.1016/j.celrep.2017.03.034, PMID: 28380364

[ref31] Gibbs E. Klemm E. Seiferth D. Kumar A. Ilca S. L. Biggin P. C.. (2023). Conformational transitions and allosteric modulation in a heteromeric glycine receptor. Nat. Commun. 14:1363. doi: 10.1038/s41467-023-37106-7, PMID: 36914669 PMC10011588

[ref32] Giménez C. Pérez-Siles G. Martínez-Villarreal J. Arribas-González E. Jiménez E. Núñez E.. (2012). A novel dominant hyperekplexia mutation Y705C alters trafficking and biochemical properties of the presynaptic glycine transporter GlyT2. J. Biol. Chem. 287, 28986–29002. doi: 10.1074/jbc.M111.319244, PMID: 22753417 PMC3436537

[ref33] Grenningloh G. Schmieden V. Schofield P. R. Seeburg P. H. Siddique T. Mohandas T. K.. (1990). Alpha subunit variants of the human glycine receptor: primary structures, functional expression and chromosomal localization of the corresponding genes. EMBO J. 9, 771–776. doi: 10.1002/j.1460-2075.1990.tb08172.x, PMID: 2155780 PMC551735

[ref34] Griffon N. Buttner C. Nicke A. Kuhse J. Schmalzing G. Betz H. (1999). Molecular determinants of glycine receptor subunit assembly. EMBO J. 18, 4711–4721. doi: 10.1093/emboj/18.17.4711, PMID: 10469650 PMC1171544

[ref35] Harvey R. J. Depner U. B. Wässle H. Ahmadi S. Heindl C. Reinold H.. (2004). GlyR α3: an essential target for spinal PGE_2_-mediated inflammatory pain sensitization. Science 304, 884–887. doi: 10.1126/science.1094925, PMID: 15131310

[ref36] Harvey K. Duguid I. C. Alldred M. J. Beatty S. E. Ward H. Keep N. H.. (2004). The GDP-GTP exchange factor collybistin: an essential determinant of neuronal gephyrin clustering. J. Neurosci. 24, 5816–5826. doi: 10.1523/jneurosci.1184-04.2004, PMID: 15215304 PMC6729214

[ref37] Harvey R. J. Topf M. Harvey K. Rees M. I. (2008). The genetics of hyperekplexia: more than startle! Trends Genet. 24, 439–447. doi: 10.1016/j.tig.2008.06.005, PMID: 18707791

[ref38] Huang X. Chen H. Michelsen K. Schneider S. Shaffer P. L. (2015). Crystal structure of human glycine receptor-α3 bound to antagonist strychnine. Nature 526, 277–280. doi: 10.1038/nature14972, PMID: 26416729

[ref39] Iossifov I. O'Roak B. J. Sanders S. J. Ronemus M. Krumm N. Levy D.. (2014). The contribution of *de novo* coding mutations to autism spectrum disorder. Nature 515, 216–221. doi: 10.1038/nature13908, PMID: 25363768 PMC4313871

[ref40] Jonsson S. Morud J. Pickering C. Adermark L. Ericson M. Soderpalm B. (2012). Changes in glycine receptor subunit expression in forebrain regions of the Wistar rat over development. Brain Res. 1446, 12–21. doi: 10.1016/j.brainres.2012.01.050, PMID: 22330726

[ref41] Kins S. Betz H. Kirsch J. (2000). Collybistin, a newly identified brain-specific GEF, induces submembrane clustering of gephyrin. Nat. Neurosci. 3, 22–29. doi: 10.1038/71096, PMID: 10607391

[ref42] Kirsch J. Betz H. (1998). Glycine-receptor activation is required for receptor clustering in spinal neurons. Nature 392, 717–720. doi: 10.1038/33694, PMID: 9565032

[ref43] Krashia P. Lape R. Lodesani F. Colquhoun D. Sivilotti L. G. (2011). The long activations of α2 glycine channels can be described by a mechanism with reaction intermediates ("flip"). J. Gen. Physiol. 137, 197–216. doi: 10.1085/jgp.201010521, PMID: 21282399 PMC3032374

[ref44] Krumm N. Turner T. N. Baker C. Vives L. Mohajeri K. Witherspoon K.. (2015). Excess of rare, inherited truncating mutations in autism. Nat. Genet. 47, 582–588. doi: 10.1038/ng.3303, PMID: 25961944 PMC4449286

[ref45] Kuhse J. Kuryatov A. Maulet Y. Malosio M. L. Schmieden V. Betz H. (1991). Alternative splicing generates two isoforms of the α2 subunit of the inhibitory glycine receptor. FEBS Lett. 283, 73–77. doi: 10.1016/0014-5793(91)80557-j, PMID: 1645300

[ref46] Langlhofer G. Schaefer N. Maric H. M. Keramidas A. Zhang Y. Baumann P.. (2020). A novel glycine receptor variant with startle disease affects syndapin I and glycinergic inhibition. J. Neurosci. 40, 4954–4969. doi: 10.1523/jneurosci.2490-19.2020, PMID: 32354853 PMC7326357

[ref47] Langlhofer G. Villmann C. (2016). The intracellular loop of the glycine receptor: it's not all about the size. Front. Mol. Neurosci. 9:41. doi: 10.3389/fnmol.2016.00041, PMID: 27330534 PMC4891346

[ref48] Leacock S. Syed P. James V. M. Bode A. Kawakami K. Keramidas A.. (2018). Structure/function studies of the α4 subunit reveal evolutionary loss of a GlyR subtype involved in startle and escape responses. Front. Mol. Neurosci. 11:23. doi: 10.3389/fnmol.2018.00023, PMID: 29445326 PMC5797729

[ref49] Levi S. Vannier C. Triller A. (1998). Strychnine-sensitive stabilization of postsynaptic glycine receptor clusters. J. Cell Sci. 111, 335–345. doi: 10.1242/jcs.111.3.335, PMID: 9427682

[ref50] Lin M. S. Xiong W. C. Li S. J. Gong Z. (2017). α2-glycine receptors modulate adult hippocampal neurogenesis and spatial memory. Dev. Neurobiol. 77, 1430–1441. doi: 10.1002/dneu.22549, PMID: 29057625

[ref51] Lynch J. W. (2004). Molecular structure and function of the glycine receptor chloride channel. Physiol. Rev. 84, 1051–1095. doi: 10.1152/physrev.00042.2003, PMID: 15383648

[ref52] Lynch J. W. (2009). Native glycine receptor subtypes and their physiological roles. Neuropharmacology 56, 303–309. doi: 10.1016/j.neuropharm.2008.07.034, PMID: 18721822

[ref53] Malosio M. L. Marqueze-Pouey B. Kuhse J. Betz H. (1991). Widespread expression of glycine receptor subunit mRNAs in the adult and developing rat brain. EMBO J. 10, 2401–2409. doi: 10.1002/j.1460-2075.1991.tb07779.x, PMID: 1651228 PMC452935

[ref54] Mangin J. M. Baloul M. Prado De Carvalho L. Rogister B. Rigo J. M. Legendre P. (2003). Kinetic properties of the α2 homo-oligomeric glycine receptor impairs a proper synaptic functioning. J. Physiol. 553, 369–386. doi: 10.1113/jphysiol.2003.052142, PMID: 12972628 PMC2343566

[ref55] Manzke T. Niebert M. Koch U. R. Caley A. Vogelgesang S. Hulsmann S.. (2010). Serotonin receptor 1A-modulated phosphorylation of glycine receptor α3 controls breathing in mice. J. Clin. Invest. 120, 4118–4128. doi: 10.1172/JCI43029, PMID: 20978350 PMC2964980

[ref56] Marcogliese P. C. Deal S. L. Andrews J. Harnish J. M. Bhavana V. H. Graves H. K.. (2022). *Drosophila* functional screening of *de novo* variants in autism uncovers damaging variants and facilitates discovery of rare neurodevelopmental diseases. Cell Rep. 38:110517. doi: 10.1016/j.celrep.2022.110517, PMID: 35294868 PMC8983390

[ref57] Matzenbach B. Maulet Y. Sefton L. Courtier B. Avner P. Guénet J. L.. (1994). Structural analysis of mouse glycine receptor α subunit genes. Identification and chromosomal localization of a novel variant. J. Biol. Chem. 269, 2607–2612. doi: 10.1016/s0021-9258(17)41987-9, PMID: 7507926

[ref58] McCracken L. M. Lowes D. C. Salling M. C. Carreau-Vollmer C. Odean N. N. Blednov Y. A.. (2017). Glycine receptor α3 and α2 subunits mediate tonic and exogenous agonist-induced currents in forebrain. Proc. Natl. Acad. Sci. USA 114, E7179–E7186. doi: 10.1073/pnas.1703839114, PMID: 28784756 PMC5576794

[ref59] Mendes M. Chen D. Z. Engchuan W. Leal T. P. Thiruvahindrapuram B. Trost B.. (2025). Chromosome X-wide common variant association study in autism spectrum disorder. Am. J. Hum. Genet. 112, 135–153. doi: 10.1016/j.ajhg.2024.11.008, PMID: 39706197 PMC11739886

[ref60] Meyer G. Kirsch J. Betz H. Langosch D. (1995). Identification of a gephyrin binding motif on the glycine receptor β subunit. Neuron 15, 563–572. doi: 10.1016/0896-6273(95)90145-0, PMID: 7546736

[ref61] Mir A. Song Y. Lee H. Khanahmad H. Khorram E. Nasiri J.. (2023). Whole exome sequencing revealed variants in four genes underlying X-linked intellectual disability in four Iranian families: novel deleterious variants and clinical features with the review of literature. BMC Med. Genet. 16:239. doi: 10.1186/s12920-023-01680-y, PMID: 37821930 PMC10566173

[ref62] Molchanova S. M. Comhair J. Karadurmus D. Piccart E. Harvey R. J. Rigo J. M.. (2017). Tonically active α2 subunit-containing glycine receptors regulate the excitability of striatal medium spiny neurons. Front. Mol. Neurosci. 10:442. doi: 10.3389/fnmol.2017.00442, PMID: 29375305 PMC5767327

[ref63] Morelli G. Avila A. Ravanidis S. Aourz N. Neve R. L. Smolders I.. (2017). Cerebral cortical circuitry formation requires functional glycine receptors. Cereb. Cortex 27, 1863–1877. doi: 10.1093/cercor/bhw025, PMID: 26891984

[ref64] Nishizono H. Darwish M. Endo T. A. Uno K. Abe H. Yasuda R. (2020). Glycine receptor α4 subunit facilitates the early embryonic development in mice. Reproduction 159:41. doi: 10.1530/REP-19-0312, PMID: 31689234

[ref65] Nobles R. D. Zhang C. Müller U. Betz H. Mccall M. A. (2012). Selective glycine receptor α2 subunit control of crossover inhibition between the on and off retinal pathways. J. Neurosci. 32, 3321–3332. doi: 10.1523/jneurosci.5341-11.2012, PMID: 22399754 PMC3438913

[ref66] Pilorge M. Fassier C. Le Corronc H. Potey A. Bai J. De Gois S.. (2016). Genetic and functional analyses demonstrate a role for abnormal glycinergic signaling in autism. Mol. Psychiatry 21, 936–945. doi: 10.1038/mp.2015.139, PMID: 26370147 PMC5382231

[ref67] Pinto D. Pagnamenta A. T. Klei L. Anney R. Merico D. Regan R.. (2010). Functional impact of global rare copy number variation in autism spectrum disorders. Nature 466, 368–372. doi: 10.1038/nature09146, PMID: 20531469 PMC3021798

[ref68] Piton A. Gauthier J. Hamdan F. F. Lafreniere R. G. Yang Y. Henrion E.. (2011). Systematic resequencing of X-chromosome synaptic genes in autism spectrum disorder and schizophrenia. Mol. Psychiatry 16, 867–880. doi: 10.1038/mp.2010.54, PMID: 20479760 PMC3289139

[ref69] Prior P. Schmitt B. Grenningloh G. Pribilla I. Multhaup G. Beyreuther K.. (1992). Primary structure and alternative splice variants of gephyrin, a putative glycine receptor-tubulin linker protein. Neuron 8, 1161–1170. doi: 10.1016/0896-6273(92)90136-2, PMID: 1319186

[ref70] Quan A. Robinson P. J. (2013). Syndapin – a membrane remodelling and endocytic F-BAR protein. FEBS J. 280, 5198–5212. doi: 10.1111/febs.12343, PMID: 23668323

[ref71] Rees M. I. Harvey K. Pearce B. R. Chung S. K. Duguid I. C. Thomas P.. (2006). Mutations in the gene encoding GlyT2 (*SLC6A5*) define a presynaptic component of human startle disease. Nat. Genet. 38, 801–806. doi: 10.1038/ng1814, PMID: 16751771 PMC3204411

[ref72] Rees M. I. Harvey K. Ward H. White J. H. Evans L. Duguid I. C.. (2003). Isoform heterogeneity of the human gephyrin gene (*GPHN*), binding domains to the glycine receptor, and mutation analysis in hyperekplexia. J. Biol. Chem. 278, 24688–24696. doi: 10.1074/jbc.M301070200, PMID: 12684523

[ref73] Rees M. I. Lewis T. M. Kwok J. B. Mortier G. R. Govaert P. Snell R. G.. (2002). Hyperekplexia associated with compound heterozygote mutations in the β-subunit of the human inhibitory glycine receptor (*GLRB*). Hum. Mol. Genet. 11, 853–860. doi: 10.1093/hmg/11.7.853, PMID: 11929858

[ref74] San Martin L. S. Armijo-Weingart L. Araya A. Yevenes G. E. Harvey R. J. Aguayo L. G. (2021). Contribution of GlyR α3 subunits to the sensitivity and effect of ethanol in the nucleus accumbens. Front. Mol. Neurosci. 14:756607. doi: 10.3389/fnmol.2021.756607, PMID: 34744627 PMC8570041

[ref75] San Martin L. Gallegos S. Araya A. Romero N. Morelli G. Comhair J.. (2020). Ethanol consumption and sedation are altered in mice lacking the glycine receptor α2 subunit. Br. J. Pharmacol. 177, 3941–3956. doi: 10.1111/bph.15136, PMID: 32436225 PMC7429487

[ref76] Schaefer N. Harvey R. J. Villmann C. (2022). Startle disease: new molecular insights into an old neurological disorder. Neuroscientist 29, 767–781. doi: 10.1177/10738584221104724, PMID: 35754344 PMC10623600

[ref77] Schaefer N. Kluck C. J. Price K. L. Meiselbach H. Vornberger N. Schwarzinger S.. (2015). Disturbed neuronal ER-golgi sorting of unassembled glycine receptors suggests altered subcellular processing is a cause of human hyperekplexia. J. Neurosci. 35, 422–437. doi: 10.1523/jneurosci.1509-14.2015, PMID: 25568133 PMC4287157

[ref78] Schaefer N. Roemer V. Janzen D. Villmann C. (2018). Impaired glycine receptor trafficking in neurological diseases. Front. Mol. Neurosci. 11:291. doi: 10.3389/fnmol.2018.00291, PMID: 30186111 PMC6110938

[ref79] Shiang R. Ryan S. G. Zhu Y. Z. Hahn A. F. O'Connell P. Wasmuth J. J. (1993). Mutations in the α1 subunit of the inhibitory glycine receptor cause the dominant neurologic disorder, hyperekplexia. Nat. Genet. 5, 351–358. doi: 10.1038/ng1293-351, PMID: 8298642

[ref80] Sola M. Bavro V. N. Timmins J. Franz T. Ricard-Blum S. Schoehn G.. (2004). Structural basis of dynamic glycine receptor clustering by gephyrin. EMBO J. 23, 2510–2519. doi: 10.1038/sj.emboj.7600256, PMID: 15201864 PMC449768

[ref81] Tian Q. Tong P. Chen G. Deng M. Cai T. Tian R.. (2023). *GLRA2* gene mutations cause high myopia in humans and mice. J. Med. Genet. 60, 193–203. doi: 10.1136/jmedgenet-2022-108425, PMID: 35396272 PMC9887403

[ref82] Troger J. Seemann E. Heintzmann R. Kessels M. M. Qualmann B. (2022). Spinal cord synaptic plasticity by GlyR β release from receptor fields and syndapin I-dependent uptake. J. Neurosci. 42, 6706–6723. doi: 10.1523/jneurosci.2060-21.2022, PMID: 35879097 PMC9436020

[ref83] Wang L. Li W. G. Huang C. Zhu M. X. Xu T. L. Wu D. Z.. (2012). Subunit-specific inhibition of glycine receptors by curcumol. J. Pharmacol. Exp. Ther. 343, 371–379. doi: 10.1124/jpet.112.195669, PMID: 22892339

[ref84] Wässle H. Heinze L. Ivanova E. Majumdar S. Weiss J. Harvey R. J.. (2009). Glycinergic transmission in the mammalian retina. Front. Mol. Neurosci. 2:6. doi: 10.3389/neuro.02.006.2009, PMID: 19924257 PMC2777502

[ref85] Werynska K. Gingras J. Benke D. Scheurer L. Neumann E. Zeilhofer H. U. (2021). A *Glra3* phosphodeficient mouse mutant establishes the critical role of protein kinase A-dependent phosphorylation and inhibition of glycine receptors in spinal inflammatory hyperalgesia. Pain 162, 2436–2445. doi: 10.1097/j.pain.0000000000002236, PMID: 34264571 PMC8374710

[ref86] Yager L. M. Garcia A. F. Wunsch A. M. Ferguson S. M. (2015). The ins and outs of the striatum: role in drug addiction. Neuroscience 301, 529–541. doi: 10.1016/j.neuroscience.2015.06.033, PMID: 26116518 PMC4523218

[ref87] Young T. L. Cepko C. L. (2004). A role for ligand-gated ion channels in rod photoreceptor development. Neuron 41, 867–879. doi: 10.1016/s0896-6273(04)00141-2, PMID: 15046720

[ref88] Young-Pearse T. L. Ivic L. Kriegstein A. R. Cepko C. L. (2006). Characterization of mice with targeted deletion of glycine receptor α2. Mol. Cell. Biol. 26, 5728–5734. doi: 10.1128/mcb.00237-06, PMID: 16847326 PMC1592777

[ref89] Yu H. Bai X. C. Wang W. (2021). Characterization of the subunit composition and structure of adult human glycine receptors. Neuron 109:e2706, 2707–2716.e6. doi: 10.1016/j.neuron.2021.08.019, PMID: 34473954

[ref90] Zhang Y. Bode A. Nguyen B. Keramidas A. Lynch J. W. (2016). Investigating the mechanism by which gain-of-function mutations to the α1 glycine receptor cause hyperekplexia. J. Biol. Chem. 291, 15332–15341. doi: 10.1074/jbc.M116.728592, PMID: 27226610 PMC4946944

[ref91] Zhang Y. Dixon C. L. Keramidas A. Lynch J. W. (2015). Functional reconstitution of glycinergic synapses incorporating defined glycine receptor subunit combinations. Neuropharmacology 89, 391–397. doi: 10.1016/j.neuropharm.2014.10.026, PMID: 25445488

[ref92] Zhang Y. Ho T. N. T. Harvey R. J. Lynch J. W. Keramidas A. (2017). Structure-function analysis of the GlyR α2 subunit autism mutation p.R323L reveals a gain-of-function. Front. Mol. Neurosci. 10:158. doi: 10.3389/fnmol.2017.00158, PMID: 28588452 PMC5440463

[ref93] Zhang C. Nobles R. D. McCall M. A. (2015). GlyR α2, not GlyR α3, modulates the receptive field surround of OFF retinal ganglion cells. Vis. Neurosci. 32:E026. doi: 10.1017/S0952523815000280, PMID: 26923349 PMC8982118

[ref94] Zhang X. B. Sun G. C. Liu L. Y. Yu F. Xu T. L. (2008). α2 subunit specificity of cyclothiazide inhibition on glycine receptors. Mol. Pharmacol. 73, 1195–1202. doi: 10.1124/mol.107.042655, PMID: 18162605

[ref95] Zhu H. Gouaux E. (2021). Architecture and assembly mechanism of native glycine receptors. Nature 599, 513–517. doi: 10.1038/s41586-021-04022-z, PMID: 34555840 PMC8647860

