# Supplementary material for: The emerging role of glycine receptor α2 subunit defects in neurodevelopmental disorders
Source: Front Mol Neurosci. 2025 Feb 11;18:1550863. doi: 10.3389/fnmol.2025.1550863 (PMC11850347; doi:10.3389/fnmol.2025.1550863)
Supplement: Supplementary file 1 [file Table_1.docx]

| cDNA | Precursor/ mature numbering | Inheritance, sex | Domain | Functional studies | Clinical features | Refs |
| --- | --- | --- | --- | --- | --- | --- |
| c.16G>C | p.V6L  p.V-22L | X, female | Signal peptide | Altered signal peptide cleavage, reduced cell-surface expression. Partial LOF | ASD; verbal IQ 63; non-verbal IQ 103. | 1,2 |
| c.140T>C | p.F47S  p.F20S | *De novo*, female | ECD | No functional assays to date | DD/ID; inattention / hyperactivity; sleep disturbance; strabismus, nystagmus (improved with age); epilepsy; infantile spasms and then normal interictal EEG; cortical and white-matter atrophy, including vermian atrophy. | 3 |
| c.195T>G | p.N65K  p.N38K | *De novo*, male | ECD | N-linked glycosylation defect, reduced whole-cell and cell-surface expression. Reduced I_max_ and increased glycine EC_50_. Reduced IPSC amplitudes. Partial LOF | Assigned as a ‘designated unaffected sibling’ to an ASD proband. | 2,4 |
| c.407A>G | p.N136S  p.N109S | *De novo,* male | ECD | Reduced cell-surface expression and increased glycine EC_50_. Partial LOF | ASD | 1,5 |
| c.458G>A | p.R153Q p.R126Q | *De novo,* male | ECD | R126Q abolishes critical hydrogen bonds in the glycine-binding site, leading to the destabilization of the ligand-binding region. Both whole-cell and cell-surface expression levels are reduced. Increased glycine EC_50_. Partial LOF | Non-syndromic autism; severe language delay with functional language; mild intellectual disability (verbal IQ 63, performance IQ 67 and full-scale IQ 63); and generalized tonic-clonic seizures starting at 18 years. | 5 |
| c.718A>G | p.K240E p.K213E | X, male | ECD | K213E induces clash with H208 in closed state, but makes additional contacts with Y209 in open state - predicted to favor open state. Reduced cell-surface expression. IPSCs have larger amplitudes, faster rise times and slower decay times. GOF | Refractory epilepsy; microcephaly; severe developmental delay. | 2 |
| c.754C>T | p.R252C p.R225C | X, male | ECD | Overexpression of R225C variant in pre-synaptic *Drosophila* photoreceptors decreases amplitude of "OFF" transients, indicating a decrease in synaptic transmission. LOF | DD/ID; hypotonia/incoordination; dysmorphic craniofacial features; sleep disturbance; strabismus. | 3 |
| c.777C>G | p.I259M p.I232M | *De novo*, female | TM1 | No functional assays to date | DD/ID; hypotonia/incoordination; child psychosis; sleep disturbance; mild dysmorphic features; left fronto-temporal spike waves focus, which diffuses in the right frontal region, activated by sleep; increased signal intensity in FLAIR of the subcortical white matter of the frontal region. | 3 |
| c.862G>A | p.A288T p.A261T | X, male | TM2 | No functional assays to date | DD/ID with regression; hypotonia/incoordination (ataxia); ASD; myopia; epilepsy; generalized slowing and generalized myoclonic epileptiform associated discharges with jerks; minimally increased T2 signal intensity on the occipital lobes. | 3 |
| c.887C>T | p.T296M p.T269M | *De novo*, multiple female probands | TM2 | Reduced cell-surface expression and I_max_ counterbalanced by reduced glycine EC_50_ and leakage current. AOF | DD/ID; hytotonia/incoordination; ASD; inattention/hyperactivity; sleep disturbance; microcephaly; epilepsy; various ocular defects including myopia, astigmatism and nystagmus. | 2,3,6 |
| ΔEx8-9 | p.K310Vfs4X  p.K283Vfs4X | X, male | Loss of TM3, ICD, TM4 | Undetectable cell surface expression; mislocalized to the cytoplasm. Glycine-induced currents are abolished. Total LOF | ASD; low average IQ (verbal IQ 93, performance IQ 75 and full-scale IQ 82); difficulties with motor coordination; language delay; bilateral myopia. | 5,7 |
| c.1048C>T | p.R350C p.R323C | X (maternal), male siblings | TM3-TM4 loop | No functional assays to date | Severe ID; developmental and speech delay; aggressive behavior; epilepsy; facial phenotypes, including broad face, prominent lips, low-set-ears, broad eyebrows, long and prominent eyelashes, and a broad nasal tip. | 8 |
| c.1049G>T | p.R350L p.R323L | X (maternal), female | TM3-TM4 loop | Small increase in glycine EC_50_ counterbalanced by IPSCs with slower rise and decay times in artificial synapses, as well as a longer duration of active periods and an increase in single-channel conductance. GOF | ASD; loss of acquired words; seizures; mild motor developmental delay; macrocephaly; hypothyroidism. | 9,10 |
| c.1186C>A | p.P396T p.P369T | X, male | TM3-TM4 loop | No functional assays to date | DD/ID; hypotonia/incoordination; ASD; myopia, astigmatism. | 3 |
| c.1199C>T | p.P400L p.P373L | X, male | TM3-TM4 loop | No functional assays to date | DD/ID; ASD; inattention/hyperactivity; epilepsy; mild dysmorphic features; right temporal focus of high and polymorphic alpha spike-wave complexes with ipsilateral propagation; increased signal intensity in FLAIR of the cortical matter of the parietal region. | 3 |
| c.1334G>A | p.R445Q p.R418Q | X, male | ECD (C-terminus) | No functional assays to date | DD/ID; hypotonia/incoordination (impaired fine motor coordination); suspected ASD; inattention/hyperactivity; sleep disturbance; reduced visual acuity; epilepsy; left and right posterior and right frontal intermittent slowing, bilateral polyspikes during sleep, and excessive beta-activity (with medications). | 3 |

**Supplementary Table 1: GlyR α2 subunit missense variants in neurodevelopmental disorders**

Key: WT = wild type; AA = amino acid; ECD = extracellular domain; TM = transmembrane; IPSC = inhibitory postsynaptic current; LOF = loss-of-function; GOF = gain-of-function; AOF = alteration-of-function; ASD = autism spectrum disorder; DD = developmental delay; ID = intellectual disability.

**References**

1. Iossifov, I., O'Roak, B.J., Sanders, S.J., Ronemus, M., Krumm, N., Levy, D., *et al* (2014). The contribution of *de novo* coding mutations to autism spectrum disorder. *Nature* 515, 216–221. doi: 10.1038/nature13908

2. Chen, X., Wilson, K.A., Schaefer, N., De Hayr, L., Windsor, M., Scalais, E., *et al* (2022) Loss, gain and altered function of GlyR α2 subunit mutations in neurodevelopmental disorders. *Front. Mol. Neurosci.* 15, 886729. doi: 10.3389/fnmol.2022.886729

3. Marcogliese, P.C., Deal, S.L., Andrews, J., Harnish, J.M., Bhavana, V.H., Graves, H.K., *et al* (2022). Drosophila functional screening of *de novo* variants in autism uncovers damaging variants and facilitates discovery of rare neurodevelopmental diseases. *Cell Rep*. 38, 110517. doi: 10.1016/j.celrep.2022.110517

4. Krumm, N., Turner, T.N., Baker, C., Vives, L., Mohajeri, K., Witherspoon, K., *et al* (2015) Excess of rare, inherited truncating mutations in autism. *Nat. Genet*. 47, 582–588. doi: 10.1038/ng.3303

5. Pilorge, M., Fassier, C., Le Corronc, H., Potey, A., Bai, J., De Gois, S., *et al* (2016) Genetic and functional analyses demonstrate a role for abnormal glycinergic signaling in autism. *Mol. Psychiatry* 21, 936–945. doi: 10.1038/mp.2015.139

6. Deciphering Developmental Disorders Study (2017) Prevalence and architecture of *de novo* mutations in developmental disorders. *Nature* 542, 433–438. doi: 10.1038/nature21062

7. Pinto, D., Pagnamenta, A.T., Klei, L., Anney, R., Merico, D., Regan, R., *et al* (2010) Functional impact of global rare copy number variation in autism spectrum disorders. *Nature* 466, 368–372. doi: 10.1038/nature09146

8. Mir, A., Song, Y., Lee, H., Khanahmad, H., Khorram, E., Nasiri, J., Tabatabaiefar, M.A. (2023) Whole exome sequencing revealed variants in four genes underlying X-linked intellectual disability in four Iranian families: novel deleterious variants and clinical features with the review of literature. *BMC Med. Genomics* 16, 239. doi: 10.1186/s12920-023-01680-y

9. Zhang, Y., Ho, T.N.T., Harvey, R.J., Lynch, J.W., Keramidas, A. (2017) Structure-function analysis of the GlyR α2 subunit autism mutation p.R323L reveals a gain-of-function. *Front. Mol. Neurosci.* 10, 158. doi: 10.3389/fnmol.2017.00158

10. Piton, A., Gauthier, J., Hamdan, F.F., Lafreniere, R.G., Yang, Y., Henrion, E., *et al* (2011) Systematic resequencing of X-chromosome synaptic genes in autism spectrum disorder and schizophrenia. *Mol. Psychiatry* 16, 867–880. doi: 10.1038/mp.2010.54
